# Predictive accuracy of diaphragm ultrasound and diaphragmatic rapid shallow breathing index combined assessment for successful weaning in neurocritical patients on mechanical ventilation

**DOI:** 10.3389/fneur.2026.1775196

**Published:** 2026-04-10

**Authors:** Liping Liao, Ying Gao, Lei Xu, Peng Chen, Ke Wang, Yongbing Deng, Xiaosu Wu, Cui Huang

**Affiliations:** 1 Department of Neurosurgery, Chongqing Emergency Medical Center, Chongqing, China; 2 School of Nursing, Chengdu University of Traditional Chinese Medicine, Chengdu, Sichuan, China; 3 Department of Nursing, Chongqing Emergency Medical Center, Chongqing, China

**Keywords:** diaphragmatic rapid shallow breathing index, diaphragm ultrasound, mechanical ventilation, severe neurological, weaning machine

## Abstract

**Objective:**

This study explores the predictive value of diaphragm ultrasound combined with the Diaphragmatic Rapid Shallow Breathing Index (D-RSBI) in determining weaning outcomes for patients with severe neurological conditions on mechanical ventilation.

**Methods:**

A retrospective analysis was conducted on 128 patients admitted to the Department of Neurosurgery, Chongqing Emergency Medical Center, who required mechanical ventilation. Based on weaning outcomes, patients were categorized into successful (*n* = 86) and failed (*n* = 42) weaning groups. We compared diaphragm ultrasound parameters (the main outcome indicators) before weaning, including Diaphragmatic Excursion (DE) Diaphragmatic Thickening Fraction (DTF), D-RSBI (RR/DE), as well as secondary outcome indicators mechanical ventilation time, respiratory rate (RR), and heart rate between the two groups.

**Results:**

The results revealed that the traditional RSBI (RR/tidal volume) and D-RSBI in the successful weaning group at the end of mechanical ventilation were lower than those in the failed weaning group, while DE and DTF were higher, the differences between the two groups were statistically significant (*p* < 0.05). The mechanical ventilation time in the successful weaning group was significantly shorter than that in the failed weaning group, and the difference was statistically significant (*p* < 0.05). The pre-ventilation respiratory rate and heart rate in the failed weaning group were higher than those in the successful weaning group, and the difference was statistically significant (*p* < 0.05). Multivariate logistic regression analysis identified high DE and DTF as protective factors, while low D-RSBI was a risk factor for weaning failure (p < 0.05). ROC curve analysis indicated that the combined use of diaphragm ultrasound parameters (DE, DTF) and D-RSBI for predicting successful weaning had an area under the curve (AUC) of 0.887, with 0.895 specificity and 0.862 sensitivity. This combined approach outperformed individual predictors, such as DE (AUC = 0.720), DTF (AUC = 0.786), and D-RSBI (AUC = 0.772).

**Conclusion:**

In conclusion, diaphragm ultrasound parameters combined with D-RSBI effectively predict weaning outcomes in neurocritical patients on mechanical ventilation. This combined approach shows promising predictive value but requires external validation.

## Introduction

Severe neurological patients often have severe disturbance of consciousness due to severe central nervous system dysfunction, which leads to a decrease in airway protection, and is prone to aspiration, pulmonary infection and even death (1). In the neurological intensive care unit (NICU), mechanical ventilation is an effective treatment for patients with severe neurological diseases. However, with the prolongation of mechanical ventilation time, complications such as subsequent dysfunction and ventilator-associated air pressure injury will make it difficult for patients to withdraw (2). Studies have shown that (3), even if the primary disease of respiratory failure is alleviated, 20–30% of patients will have difficulty in weaning. Respiratory muscle dysfunction is one of the main factors leading to weaning failure. Mechanical ventilation can change the structure and function of the diaphragm, causing abnormal diaphragm function, leading to treatment failure, seriously affecting the prognosis of patients, resulting in increased retention time and mortality in NICU (4). Therefore, early detection and treatment of diaphragmatic muscle injury caused by severe cerebral ischemia is of great significance for reducing NICU retention time, improving prognosis and reducing mortality.

In recent years, a large number of studies have shown that (5, 6), ultrasound, as a non-invasive, non-radiative and convenient evaluation method, can observe the motor function and morphological changes of diaphragm in real time and dynamically, and has become an important tool for evaluating the structure and function of diaphragm. In patients with mechanical ventilation, diaphragmatic ultrasound provides a more comprehensive basis for weaning evaluation (7). Diaphragm rapid shallow breathing index (D-RSBI) is a derivative index of diaphragm ultrasound detection, which can reflect the balance between respiratory power consumption and diaphragm function during weaning (8). Studies have shown that (9), D-RSBI has better sensitivity and specificity, and has been proved to be used to predict the weaning outcome of patients with mechanical ventilation. However, the value of diaphragmatic ultrasound index as a predictor of the success or failure of mechanical ventilation weaning has not been widely evaluated and promoted in clinical practice. Simply using D-RSBI for weaning assessment may ignore the contribution of auxiliary respiratory muscles to ventilation, thus affecting the accuracy of weaning prediction. Therefore, in order to verify the predictive efficacy of diaphragmatic excursion (DE) combined with D-RSBI, this study further explored the value of diaphragmatic ultrasound combined with D-RSBI in predicting the weaning outcome of patients with severe neurological mechanical ventilation, and provided a basis for clinical weaning decision-making.

## Patients and methods

### Study design

A total of 128 patients with severe mechanical ventilation in neurosurgery admitted to Department of Neurosurgery, Chongqing Emergency Medical Center were retrospectively selected as the research objects. This study was endorsed by Chongqing Emergency Medical Center and patient informed consent was exempted (No. 2024-68).

Inclusion criteria: (1) All enrolled patients were ≥18 years old and <80 years old; (2) All patients were clinically diagnosed as severe neurological diseases (such as cerebral hemorrhage, cerebral infarction, craniocerebral trauma, etc.); (3) All patients received mechanical ventilation for more than 48 h; (4) All patients had complete clinical data and ultrasound evaluation data.

Exclusion criteria: (1) patients with chronic respiratory diseases or neuromuscular diseases; (2) patients with closed thoracic drainage, pneumothorax or mediastinal emphysema; (3) Patients with incomplete or inaccessible ultrasound evaluation data.

Weaning criteria: (1) the primary disease or predisposing factors leading to mechanical ventilation were basically controlled or improved; (2) The consciousness is basically clear; (3) Hemodynamic indexes were stable; (4) Ability to breathe and cough spontaneously; (5) oxygenation index > 150 ~ 200 mmHg (1 mmHg = 0.133 kPa), positive end-expiratory pressure ≤ 5 ~ 8 cmH_2_O (1 cmH_2_O = 0.098 kPa), inhaled oxygen concentration 7.257.

Sample size calculation: Based on the Events Per Variable (10EPV) algorithm, six risk factors were identified. With a 50% success rate in weaning, the sample size is calculated as 6 × 10/50% = 120 cases, indicating that this study requires at least 120 enrolled patients.

### Methods

#### Intervention methods

Spontaneous breathing trial (SBT) was performed on patients who met the above criteria. The mode of synchronous intermittent mandatory ventilation-continuous positive airway pressure-weaning was adopted. The low-level pressure support was set at 8 cmH_2_O, and the expiratory end positive pressure was set at 5 cmH_2_O.

#### Diaphragm ultrasound examination method

Diaphragm ultrasound examination at the end of SBT. All patients underwent ultrasound examination in a semi-recumbent position with the head of the bed raised by 30°. Diaphragmatic excursion (DE) was measured using a 3–5 MHz convex array probe. The probe was placed at the junction of the midclavicular line or the anterior axillary line of the patient and the lower edge of the costal arch. The liver or spleen was used as the diaphragm sound transmission window. The probe pointed to the head and back, so that the sound beam reached and perpendicular to the middle and posterior 1/3 of the diaphragm. On the basis of the ideal two-dimensional image, M-ultrasound was used to display the diaphragm movement. The M-ultrasound sampling line pointed to the top of the diaphragm and the angle with the long axis <30° to obtain the maximum diaphragm mobility. Diaphragm thickness measurement: The patient was placed in a semi-recumbent position, and the head of the bed was raised by 30°. A linear array probe with a frequency of 10 MHz was placed in the 8th to 10th intercostal space of the anterior axillary line or the midaxillary line, and the diaphragm structure at the costal angle was displayed perpendicular to the chest wall. Based on the ideal two-dimensional image, the thickness of the diaphragm at 0.5–2.0 cm below the costophrenic angle was measured using the B-type or M-type mode. Diaphragm thickness at end-inspiratory and end-expiratory was measured and the diaphragm thickening fraction (DTF) was calculated. DTF = (diaphragmatic end-inspiratory thickness-diaphragmatic end-expiratory thickness)/diaphragmatic end-expiratory thickness. Instrument: Edge II ultrasonic diagnostic apparatus (Sonosite, United States); servo-i ventilator (Maquet, Germany).

#### Grouping methods

Grouped according to the success or failure of weaning from the ventilator. Criteria for success and failure of mechanical ventilation withdrawal: Using the standard assessment screening for mechanical ventilation withdrawal, if within 2 min of the SBT, the following conditions occur: ① The ratio of respiratory rate to tidal volume (respiratory rate/tidal volume) is greater than 105; ② The respiratory rate is less than 8 breaths per minute or more than 35 breaths per minute; ③ The tidal volume of spontaneous breathing is less than 4 mL/kg; ④ The heart rate is greater than 140 beats per minute or changes by more than 20%, and new arrhythmia occurs; ⑤ The arterial blood oxygen saturation is less than 90%. Then, mechanical ventilation withdrawal is considered a failure. Through the 2-min SBT, if the patient can continue spontaneous breathing for 30 to 120 min, it is determined as successful mechanical ventilation withdrawal; otherwise, it is still considered a failure.

#### Methods of data collection

The age, gender, body mass index (BMI), acute physiology and chronic health evaluation II (APACHE II) score, primary disease (cerebral hemorrhage, cerebral infarction, craniocerebral trauma), parameters of SBT before weaning (respiration, heart rate, oxygenation index, tidal volume), mechanical ventilation time, blood gas analysis index [arterial partial pressure of carbon dioxide (PaCO_2_), arterial partial pressure of oxygen/fraction of inspired oxygen (PaO_2_/FiO_2_)], diaphragmatic ultrasound index [DE, diaphragmatic thickening fraction (DTF), D-RSBI)] of the two groups were collected. D-RSBI = (respiratory rate (RR)/Diaphragmatic Excursion (DE). DTF = (diaphragmatic end-inspiratory diaphragmatic thickness-diaphragmatic end-respiratory diaphragmatic thickness)/diaphragmatic end-respiratory diaphragmatic thickness.

### Statistical methods

SPSS 27.0 statistical software was used for data analysis. The mean of measurement data was expressed as (mean ± SD), and *t*-test was used for comparison between groups. Qualitative data were expressed in *n* (%), and chi-square (*χ*^2^) test was performed for comparison between groups. Multivariate Logistic regression analysis was used to analyze the risk factors affecting the weaning outcome of neurocritical patients, and the receiver operating characteristic curve (ROC) was drawn. The area under the curve (AUC), specificity and sensitivity were used to evaluate the predictive value. The comparison of AUC values adopts the DeLong test. *p* < 0.05 was considered statistically significant.

This study explores the relationship between D-RSBI and the weaning process of neurocritical care patients on mechanical ventilation. The cases group consists of patients who failed the weaning process, while the control group consists of patients who successfully weaned. The D-RSBI score for the successful weaning group was 1.65 ± 0.27, and for the failed weaning group it was 2.02 ± 0.40. With a bilateral *α* of 0.05 and a test power of 90%, using the sample size calculation formula for case–control studies, it was calculated that at least 114 cases were needed. The sample size included in this study was in line with the calculation results.

## Results

### Baseline characteristics analysis

**Figure 1:**
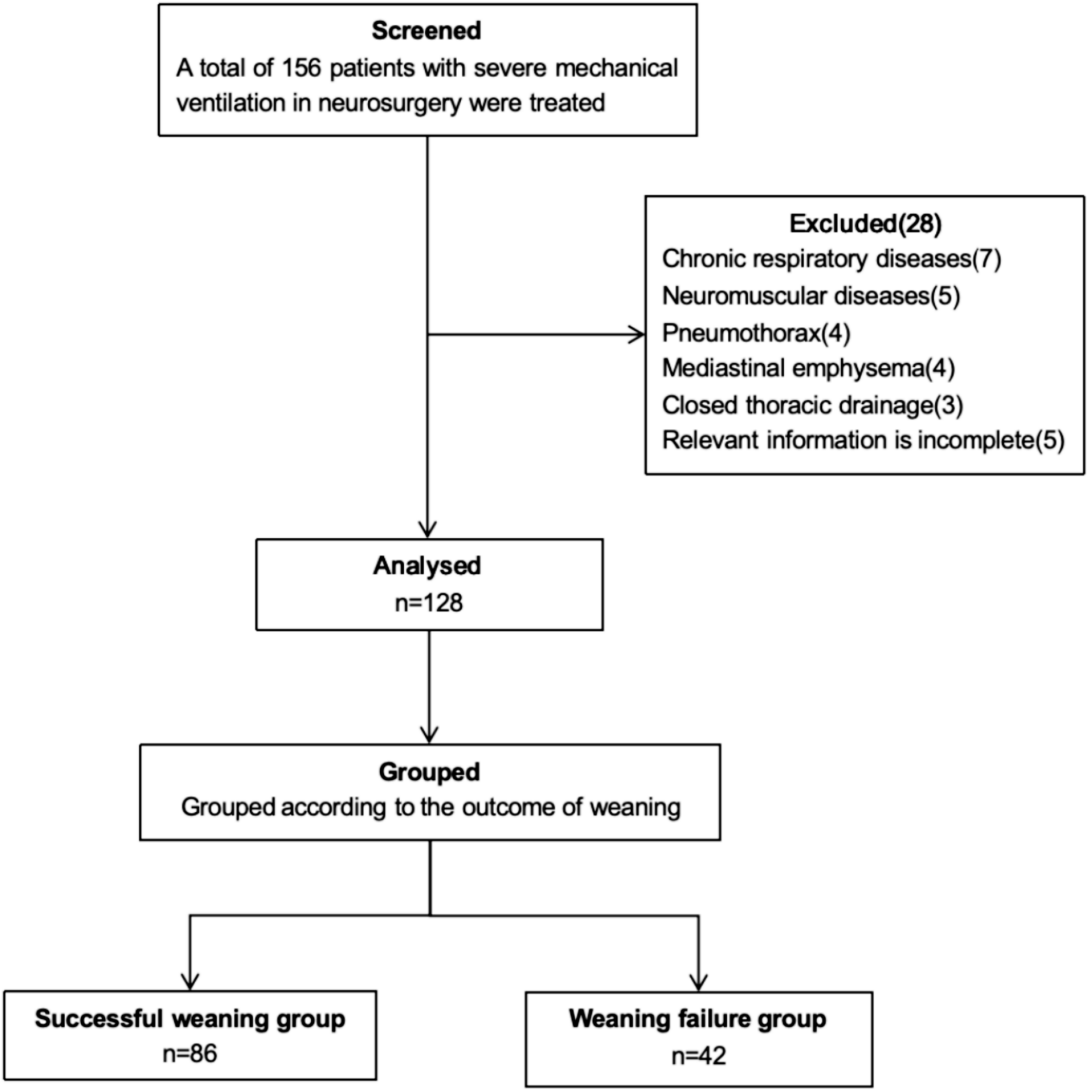
Flow chart of patient screening.

A total of 128 patients with severe neurological diseases were included, including 86 cases (67.19%) of successful weaning and 42 cases (32.81%) of failed weaning. There was no significant difference in age, gender, BMI, APACHE II score, original diseases and blood gas analysis between the successful weaning group and the failure weaning group (*p* > 0.05). The mechanical ventilation time in the successful weaning group was significantly shorter than that in the failure weaning group, and the difference was statistically significant (*p* < 0.05) (see Table 1). The flow chart of patient screening is shown in Figure 1.

**Table 1:** Baseline characteristics analysis [*n*(%), (mean ± SD)].

Index	Successful weaning group (*n* = 86)	Weaning failure group (*n* = 42)	*χ^2^/t*	*P*
Age (years)	65.71 ± 4.32	66.88 ± 4.57	1.413	0.160
Gender			0.184	0.668
Male	54(62.79)	28(66.67)		
Female	32(37.21)	14(33.33)		
BMI (kg/m^2^)	23.49 ± 2.47	22.85 ± 2.56	1.346	0.181
APACHEII score	16.33 ± 3.25	17.29 ± 3.17	1.581	0.116
Original diseases			1.185	0.874
Intracranial bleeding	12(13.95)	4(9.52)		
Cerebral infarction	14(16.28)	5(11.90)		
Craniocerebral trauma	16(18.61)	7(16.67)		
Heart failure	11(12.79)	8(19.05)		
Severe pneumonia	19(22.09)	11(26.19)		
Miscellaneous	14(16.28)	7(16.67)		
PaO_2_ (mmH/g)	40.48 ± 3.52	39.45 ± 3.67	1.525	0.130
PaO_2_/FiO_2_ (mmH/g)	254.39 ± 16.29	259.45 ± 19.41	1.547	0.124
Mechanical ventilation time (d)	4.67 ± 0.58	4.92 ± 0.42	2.412	0.017

BMI, body mass index; APACHEII score, acute physiology and chronic health evaluation II score; PaCO_2,_ arterial partial pressure of carbon dioxide; PaO_2_/FiO_2_, arterial partial pressure of oxygen/fraction of inspired oxygen.

### Comparison of pre-SBT parameters between the two groups

The respiration and heart rate before SBT in the weaning failure group were higher than those in the weaning success group, and the difference between the two groups was statistically significant (*p* < 0.05) (see Table 2).

**Table 2:** Parameter analysis of two groups of patients before SBT test (mean ± SD).

Index	Successful weaning group (*n* = 86)	Weaning failure group (*n* = 42)	*t*	*P*
Breathing (times/min)	16.58 ± 2.83	19.79 ± 2.95	5.930	<0.001
Heart rate (beats/min)	81.35 ± 5.48	92.62 ± 6.27	10.415	<0.001
Oxygenation index	294.38 ± 53.59	292.31 ± 54.80	0.204	0.839
Tidal volume (mL)	468.97 ± 89.39	436.24 ± 85.75	1.971	0.051

### Comparison of parameters at the end of SBT test between the two groups of patients

Respiratory, RSBI and D-RSBI at the end of SBT in the successful weaning group were lower than those in the failure weaning group, DE and DTF were higher than those in the failure weaning group, and the difference between the two groups was statistically significant (*p* < 0.05) (see Table 3).

**Table 3:** Analysis of parameters at the end of SBT test in the two groups of patients (mean ± SD).

Index	Successful weaning group (*n* = 86)	Weaning failure group (*n* = 42)	*t*	*P*
Breathing (times/min)	16.87 ± 3.57	19.64 ± 4.52	3.771	<0.001
Heart rate (beats/min)	86.26 ± 11.74	89.52 ± 12.65	1.441	0.152
Oxygenation index	289.28 ± 48.43	287.14 ± 41.69	0.245	0.807
Tidal volume (mL)	458.58 ± 87.62	487.90 ± 93.19	1.741	0.084
RSBI	56.36 ± 7.67	60.55 ± 6.73	3.013	0.003
DE (mm)	16.83 ± 3.78	14.02 ± 2.68	4.823	<0.001
DTF (%)	32.11 ± 3.64	28.14 ± 3.69	5.768	<0.001
D-RSBI (times·min^−1^·min^−1^)	1.65 ± 0.27	2.02 ± 0.40	5.318	<0.001

RSBI, rapid shallow breathing index; DE, diaphragmatic excursion; DTF, diaphragm thickening fraction; D-RSBI, diaphragmatic rapid shallow breathing index.

### Analysis of risk factors affecting weaning outcome

Univariate analysis showed that mechanical ventilation time, RSBI, DE, DTF and D-RSBI were the influencing factors of weaning failure (*p* < 0.05). Taking the success of weaning as the dependent variable (yes = 1, no = 0), the mechanical ventilation time, RSBI, DE, DTF and D-RSBI were input with the actual value. The multicollinearity diagnostics results show that the VIF values for breathing, mechanical ventilation time, RSBI, DE, DTF, and D-RSBI are 1.048, 1.057, 1.105, 1.126, 1.095, and 1.071, respectively. After controlling for confounding factors (APACHE II, cause of neurological injury, level of consciousness (GCS), sedation status, use of neuromuscular blockers), the multivariate analysis revealed that high DE and high DTF were protective factors for mechanical ventilation weaning failure in neurocritical patients (*p* < 0.05), and low D-RSBI was a risk factor for mechanical ventilation weaning failure in neurocritical patients (*p* < 0.05) (see Table 4). Hosmer–Lemeshow *χ*^2^ = 8.973, *p* = 0.345.

**Table 4:** Logistic regression analysis of the effect of mechanical ventilation weaning outcome in patients with severe neurological diseases.

Factors	*β*	SE	Wald *χ*^2^	*P*	OR(95%CI)
Breathing	0.072	0.043	2.895	0.084	1.143(1.165 ~ 1.839)
Mechanical ventilation time	0.929	0.525	3.124	0.077	2.532(0.904 ~ 7.091)
RSBI	0.065	0.039	2.742	0.098	1.067(0.988 ~ 1.153)
DE	−0.201	0.085	5.598	0.018	0.818(0.692 ~ 0.966)
DTF	−0.247	0.070	12.534	<0.001	0.781(0.681 ~ 0.896)
D-RSBI	3.145	0.808	15.143	<0.001	23.223(4.764 ~ 113.216)

RSBI, rapid shallow breathing index; DE, diaphragmatic excursion; DTF, diaphragm thickening fraction; D-RSBI, diaphragmatic rapid shallow breathing index.

### The predictive efficacy of diaphragmatic ultrasound parameters DE, DTE combined with D-RSBI on weaning outcome was analyzed

**Figure 2:**
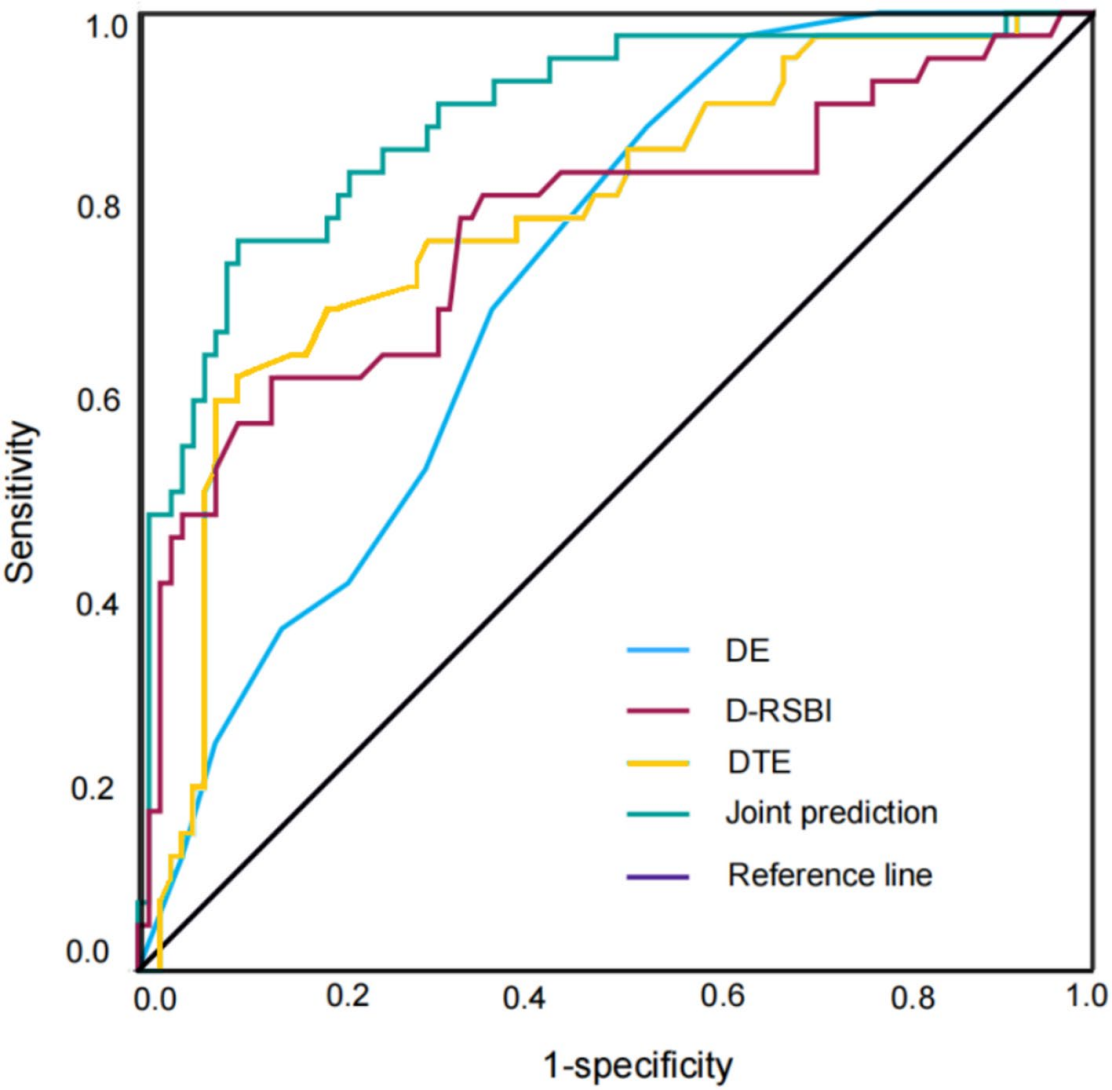
ROC curve for predicting successful weaning. ROC: receiver operating characteristic curve.

The results of ROC curve analysis showed that the AUC of diaphragmatic ultrasound parameters (DE, DTF) combined with D-RSBI in predicting the successful weaning of patients with severe neurological mechanical ventilation was 0.887, the specificity was 0.895, and the sensitivity was 0.862. The DeLong test results showed that the combined prediction was superior to the individual predictions of DE (0.720), DTF (0.786), and D-RSBI (0.772) (see Table 5 and Figure 2).

**Table 5:** Analysis of the predictive value of diaphragmatic ultrasound parameters DE, DTE combined with D-RSBI for successful weaning.

Index	AUC	95%CI	Specificity	Sensitivity	Youden index	Cutoff value
DE	0.720	0.633 ~ 0.807	0.765	0.781	0.346	17.1
DTF	0.786	0.700 ~ 0.873	0.885	0.719	0.514	30.5
D-RSBI	0.772	0.677 ~ 0.866	0.860	0.783	0.480	1.65
Joint prediction	0.887	0.824 ~ 0.950	0.895	0.862	0.657	–

AUC, area under the curve.

## Discussion

Mechanical ventilation is the key technology of respiratory support for critically ill patients, because it can provide stable respiratory support for patients who cannot breathe spontaneously, so as to maintain vital signs and ensure respiratory function. However, the use of mechanical ventilation also brings the important problem of offline timing. Early or delayed weaning may increase the risk of weaning failure, prolong hospital stay, affect the rehabilitation process, induce complications, and even increase mortality (10). In recent years, the importance of diaphragmatic function assessment in the process of weaning has been paid more and more attention by clinicians. A comprehensive assessment of diaphragmatic function is crucial before weaning attempts. Among the predictors of weaning from mechanical ventilation, the traditional RSBI has attracted much attention (11). RSBI is the ratio of respiratory rate to tidal volume, which is used to reflect the overall work ability of respiratory muscles and predict the outcome of weaning (12). However, the application of RSBI has limitations. For example, diaphragmatic atrophy may lead to decreased respiratory muscle function, which in turn affects the accuracy of RSBI (13). In the early stages of diaphragmatic atrophy, RSBI may not accurately predict weaning outcomes. Therefore, it is recommended to combine other indicators for comprehensive evaluation, such as D-RSBI, DE and DTF. These indicators can more fully reflect the diaphragm function, thereby improving the accuracy of weaning prediction. Hiding and accurately predicting the patient’s weaning time is of great clinical significance for successful weaning.

A total of 128 patients with severe neurological diseases were included in this study. The patients were grouped according to the results of weaning. Among them, 86 cases (67.19%) were successful and 42 cases (32.81%) were failed. The 67.19% success rate of weaning in this study showed that most critically neurological patients could successfully withdraw from mechanical ventilation, but about one-third of patients failed to withdraw. The failure of weaning is related to many factors, such as the complexity of the patient’s condition, complications, timing of treatment, etc. The most important factor is diaphragmatic dysfunction (14). Even if the time of receiving positive pressure ventilation is very short, the diaphragmatic contraction ability will decrease or atrophy, which will lead to diaphragmatic fatigue. The maintenance time of mechanical ventilation is negatively correlated with the decrease of diaphragmatic strength, and is affected by potential factors such as primary disease and severity (15). By comparing the clinical data of the two groups before weaning, it was found that there was no significant difference in the other observation indexes except the mechanical ventilation time (the weaning success group was shorter than the weaning failure group). This is because the longer the ventilation time, the higher the incidence of ventilator-related complications, and long-term mechanical ventilation is also likely to lead to ventilator dependence, so the longer the time, the greater the possibility of weaning failure.

In this study, it was found during SBT that the respiratory rate and heart rate before SBT in the weaning failure group were higher than those in the weaning success group, indicating that the increase of respiratory rate and heart rate may be a potential risk factor for weaning failure. This phenomenon may be related to poor basic cardiopulmonary function, strong stress response or other complications in patients with weaning failure. In addition, through the combination of diaphragmatic ultrasound parameters DE, DTF and D-RSBI, the tidal volume was replaced by DE and DTF to eliminate the interference of related auxiliary respiratory muscles. The results showed that the respiration, RSBI and D-RSBI in the successful weaning group were lower than those in the failed weaning group at the end of SBT test, and the DE and DTF in the successful weaning group were higher than those in the failed weaning group. The above results indicate that the respiratory burden of patients with weaning failure is heavier, and higher RSBI and D-RSBI indicate worse diaphragm function, which increases the risk of weaning failure. DE and DTF are important indicators of diaphragmatic function. Lower DE and DTF suggest insufficient diaphragmatic contractility, which is a potential risk factor for weaning failure (16). Some studies (17) believed that the predictive value of RSBI for weaning outcome was variable, and many studies (18, 19) found that the cut-off value of RSBI for predicting weaning outcome had great variability. However, multivariate logistic regression analysis in this study showed that RSBI was not a risk factor for mechanical ventilation failure in neurocritical patients, which may be related to the sample size included in the study and the heterogeneity of the patient population. High DE and DTF were protective factors for weaning failure of mechanical ventilation in patients with severe neurological diseases (*p* < 0.05), which indicated that higher diaphragm activity and thickening rate reflected good diaphragm function and helped to improve the probability of successful weaning. Low D-RSBI was a risk factor for weaning failure of mechanical ventilation in patients with severe neurological diseases (*p* < 0.05). D-RSBI combines diaphragmatic function and respiratory rate to accurately reflect the patient’s respiratory status and weaning risk (20).

The DE and DTF of patients with mechanical ventilation were dynamically observed by ultrasound, which could better predict the timing of weaning. In this study, the DD of patients with severe neurological mechanical ventilation was monitored by diaphragm ultrasound. It was found that the DE after the SBT test in the successful weaning group was higher than that in the failure weaning group. The optimal cut-off value (Cutoff value) showed by ROC curve analysis was 17.1 mm. The AUC predicted alone was 0.720, the specificity was 0.765, and the sensitivity was 0.781, indicating that DE can be used as an important diaphragm function index, which can provide valuable reference for weaning decision-making in patients with severe neurological diseases. Studies (21) have shown that DTF between 28 and 36% has good sensitivity and specificity in predicting the success of weaning in patients with mechanical ventilation. Gursel et al. (22) believed that when DTF < 20%, it is diaphragmatic dysfunction. In this study, the DTF of the successful weaning group was significantly higher than that of the failed weaning group. The Cutoff value of the ROC curve analysis was 30.5%, the AUC of the single prediction was 786, the specificity was 0.885, and the sensitivity was 0.719, further indicating that DTF is a more reliable ultrasound index for evaluating diaphragmatic function and can be used to predict the weaning outcome. This is mainly due to the fact that diaphragm function is related to inspiratory thickening. DTF reflects diaphragm contraction by measuring the change rate of inspiratory and expiratory thickness, so as to evaluate the performance of respiratory muscle work. More and more research centers are exploring the optimal cut-off value for evaluating the weaning failure of D-RSBI. Song et al. (8) reported that the optimal cut-off value of D-RSBI for weaning failure was 1.38 times·min-1·min-1 in 110 patients. In this study, the Cutoff value of D-RSBI was 1.65 times·min-1·min-1, the AUC predicted alone was 0.772, the specificity was 0.860, and the sensitivity was 0.783. D-RSBI is an effective weaning risk assessment tool. Lower D-RSBI values usually indicate good diaphragmatic function and a higher likelihood of successful weaning (23). In addition, the AUC of DE, DTF combined with D-RSBI in predicting the successful weaning of patients with severe mechanical ventilation in this study was 0.887, the specificity was 0.895, and the sensitivity was 0.862, which was better than that of DE, DTF and D-RSBI alone. This indicates that the combined evaluation of DE, DTF and D-RSBI can more comprehensively reflect the diaphragm function and respiratory status of patients, and thus more accurately predict the success of weaning. This joint prediction method provides clinicians with a more reliable decision-making basis for weaning, which helps to optimize the weaning strategy and reduce the risk of weaning failure.

The innovation of this study is to combine diaphragm ultrasound technology with D-RSBI to predict the weaning outcome of patients with severe neurological mechanical ventilation. This joint evaluation method can more fully reflect the balance between diaphragmatic function and respiratory load, thereby improving the accuracy of weaning prediction. The introduction of ultrasonic indicators DE and DTF provides a non-invasive, dynamic and repeatable evaluation method for clinical practice, which makes up for the limitations of weaning parameters. However, this study still has certain limitations. Firstly, the research sample comes from a single center with a limited sample size. As it is a single-center study, it lacks external validation. The retrospective analysis may affect the generalizability and extrapolation of the results. Secondly, although the combined prediction efficacy is superior to individual prediction, the study failed to fully include factors that may affect the outcome of weaning, such as the patient’s nutritional status and respiratory center drive. The adjustment for confounding factors is limited, and there may be a selection bias problem. Moreover, the operation and interpretation of diaphragm ultrasound in the study rely on the operator’s experience, which may lead to subjective bias in the results. Since the data was collected retrospectively, the reliability of the ultrasound examination could not be analyzed. Finally, the study did not explore in-depth the intervention measures after weaning failure. Future research can further optimize the combined prediction model and verify its clinical application value in multi-center, large-sample studies.

This study describes the clinical value of diaphragm ultrasound combined with D-RSBI in predicting the weaning outcome of patients with severe neurological mechanical ventilation. Our findings highlight that DE, DTF combined with D-RSBI can effectively judge the outcome of weaning, which is a potential indicator to guide the timing of weaning, and the combined prediction performance is better than that of single prediction. These results suggest that the advantages of ultrasound diaphragmatic function assessment in predicting successful weaning are increasingly valued by clinicians and have potential clinical effects.

## Data Availability

The original contributions presented in the study are included in the article/supplementary material, further inquiries can be directed to the corresponding authors.
